# Potential shared pathogenic mechanisms between endometriosis and inflammatory bowel disease indicate a strong initial effect of immune factors

**DOI:** 10.3389/fimmu.2024.1339647

**Published:** 2024-04-03

**Authors:** Haolong Zhang, Yaxin Mo, Ling Wang, Haoling Zhang, Sen Wu, Doblin Sandai, Ahmad Naqib Shuid, Xingbei Chen

**Affiliations:** ^1^ Department of Gynecology and Obstetrics, The First Affiliated Hospital of Zhejiang Chinese Medical University (Zhejiang Provincial Hospital of Chinese Medicine), Hangzhou, China; ^2^ Department of Biomedical Sciences, Advanced Medical & Dental Institute, Universiti Sains Malaysia, Penang, Malaysia; ^3^ Department of TCM Gynecology, Hangzhou TCM Hospital Affiliated to Zhejiang Chinese Medical University, Hangzhou, China

**Keywords:** endometriosis, inflammatory bowel disease, mechanisms, bioinformatics analysis, molecular pathway

## Abstract

**Introduction:**

Over the past decades, immune dysregulation has been consistently demonstrated being common charactoristics of endometriosis (EM) and Inflammatory Bowel Disease (IBD) in numerous studies. However, the underlying pathological mechanisms remain unknown. In this study, bioinformatics techniques were used to screen large-scale gene expression data for plausible correlations at the molecular level in order to identify common pathogenic pathways between EM and IBD.

**Methods:**

Based on the EM transcriptomic datasets GSE7305 and GSE23339, as well as the IBD transcriptomic datasets GSE87466 and GSE126124, differential gene analysis was performed using the limma package in the R environment. Co-expressed differentially expressed genes were identified, and a protein-protein interaction (PPI) network for the differentially expressed genes was constructed using the 11.5 version of the STRING database. The MCODE tool in Cytoscape facilitated filtering out protein interaction subnetworks. Key genes in the PPI network were identified through two topological analysis algorithms (MCC and Degree) from the CytoHubba plugin. Upset was used for visualization of these key genes. The diagnostic value of gene expression levels for these key genes was assessed using the Receiver Operating Characteristic (ROC) curve and Area Under the Curve (AUC) The CIBERSORT algorithm determined the infiltration status of 22 immune cell subtypes, exploring differences between EM and IBD patients in both control and disease groups. Finally, different gene expression trends shared by EM and IBD were input into CMap to identify small molecule compounds with potential therapeutic effects.

**Results:**

113 differentially expressed genes (DEGs) that were co-expressed in EM and IBD have been identified, comprising 28 down-regulated genes and 86 up-regulated genes. The co-expression differential gene of EM and IBD in the functional enrichment analyses focused on immune response activation, circulating immunoglobulin-mediated humoral immune response and humoral immune response. Five hub genes (SERPING1、VCAM1、CLU、C3、CD55) were identified through the Protein-protein Interaction network and MCODE.High Area Under the Curve (AUC) values of Receiver Operating Characteristic (ROC) curves for 5hub genes indicate the predictive ability for disease occurrence.These hub genes could be used as potential biomarkers for the development of EM and IBD. Furthermore, the CMap database identified a total of 9 small molecule compounds (TTNPB、CAY-10577、PD-0325901 etc.) targeting therapeutic genes for EM and IBD.

**Discussion:**

Our research revealed common pathogenic mechanisms between EM and IBD, particularly emphasizing immune regulation and cell signalling, indicating the significance of immune factors in the occurence and progression of both diseases. By elucidating shared mechanisms, our study provides novel avenues for the prevention and treatment of EM and IBD.

## 1 Introduction

Endometriosis (EM) is a prevalent gynecological ailment defined by the irregular growth of endometrial-like tissue beyond the uterus, including the ovaries, fallopian tubes, uterine surface, and peritoneum. It is typical symptoms include dysmenorrhea, chronic pelvic ache, infertility, and sexual dysfunction (1). Globally, an estimated 2%-10% of women at reproductive age had undergone endometriosis, whereby 5-21% of these women had reported severe pelvic pain. In infertility cases, this rate could escalate to 50%, meanwhile the probability of ovarian cancer increased by 50% (2, 3). These ectopic endothelial tissues were also hormonally regulated during the menstrual cycle, resulting in cyclical bleeding and inflammatory responses in the ectopic location. Researches has demonstrated that EM patients have an abnormal immune system, characterized by a paradoxical immune response towards their own tissues and anomalous secretion of inflammatory factors (4, 5). These atypical responses contribute to the persistence of inflammation and consequential tissue damage within endometriotic foci. Additionally, patients with EM may have an immune regulation imbalance, specifically impaired regulatory T-cell function. Nonetheless, comprehensive investigations are imperative to fully comprehend the intricate features and the role of endometriosis in disease progression, particularly from an immunological perspective.

Inflammatory bowel disease (IBD) is a chronic, relapsing inflammatory disease of the gastrointestinal tract, of which Crohn’s disease and ulcerative colitis are the most common subtypes (6). IBD typically presents with a variety of digestive symptoms including abdominal pain, diarrhea, weight loss and fatigue. The detailed causes and mechanisms of IBD are remain unknown, but existing research suggested that genetic, environmental and immune factors played a role in this disease (7–9). IBD usually requires life-long treatment to ease symptoms, control inflammation and maintain long-term remission. Immunological factors play a significant role in the development of IBD. The patient’s intestinal immune system exhibits aberrant responses to the ordinary intestinal flora, resulting in sustained inflammation (10, 11). Besides, cytokines, including interferon, tumor necrosis factor, and interleukin, are overexpressed in IBD, signifying immune response activation (12–14). Treatment options available include medication, nutritional support, surgical interventions and lifestyle management. And typically presents as a cyclic pattern of remission and relapse, where patients may experience symptom control for a certain period and then have acute flare-ups during another period.

EM and IBD are both chronic inflammatory conditions that affect the immune system and commonly impact young women. They share some common clinical symptoms, including abdominal pain, fatigue, infertility, menstrual irregularities, and gastrointestinal symptoms such as diarrhoea and constipation (15). Although the pathophysiology is very different, previous studies have shown that women with EM are more likely to develop IBD. And a 10-year cohort study conducted in Denmark found that women with EM have a significantly higher risk of developing IBD. The standardized incidence for Crohn’s disease was 1.5 (95% CI 1.3-1.7) and for ulcerative colitis was 1.6 (95% CI 1.3-2.0) (16). The increased risk of IBD in patients with EM was also confirmed by Lee et al. (17, 18). A Mendelian randomization study in 2023 found that EM is a risk factor for the development of IBD, especially in people with advanced EM (19). Recent research has also indicated the presence of some surprising shared immunological and molecular characteristics between EM and IBD. Lv Y’ s work shows that endometrial regenerative cells have novel anti-inflammatory and immunosuppressive effects that reduce colitis symptoms in mice, suggesting that these unique cells may have potential as a therapeutic tool for ulcerative colitis. It is important to explore this promising avenue for future therapies (20). This finding has inspired us to utilize bioinformatics tools and methods to delve deeper into the potential shared pathogenic mechanisms between these two diseases by analyzing data such as gene expression, protein interactions, pathway regulation, and more.

## 2 Materials and methods

### 2.1 Data sources and research design

**Figure 1:**
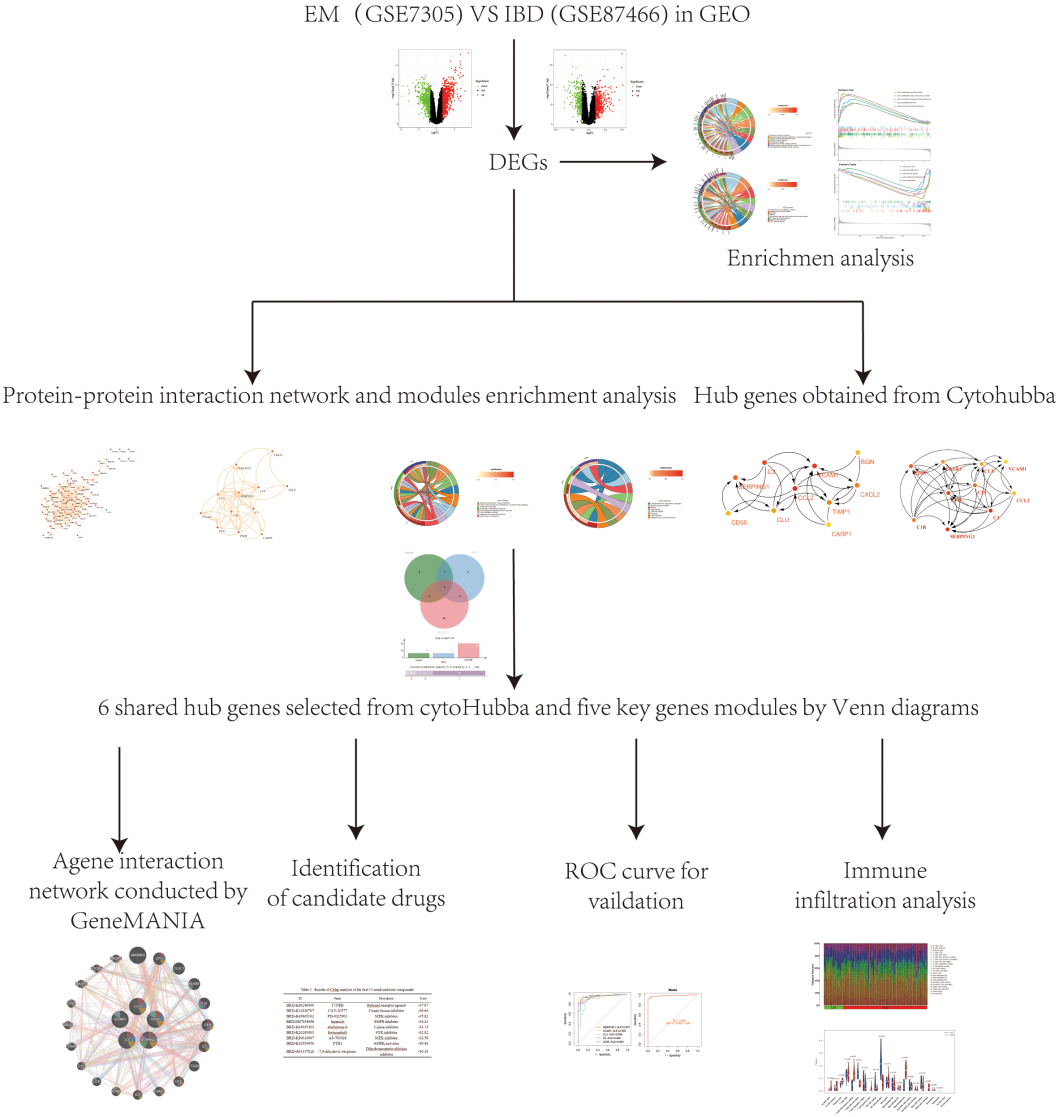
A schematic representation of the research design was created. Microarray datasets from the GEO database were used to identify co-expressed genes and their functions in EM and IBD. PPI network was systematically constructed through the STRING database, followed by the identification and filtration of key genes utilizing the MCODE tool and CytoHubba plugin within the Cytoscape platform. Subsequently, core genes relevant to EM and IBD were identified through visualization with UpSet and were further validated employing external datasets. Lastly, we used the CMap database to search for potential therapeutic small molecule compounds to reveal the shared pathogenic mechanisms of EM and IBD.

EM(GSE7305and GSE23339)and IBD(GSE87466 and GSE126124) transcriptome datasets were retrieved from Gene Expression Omnibus (GEO) (https://www.ncbi.nlm.nih.gov/geo/). The GSE7305 dataset was utilized for the Endometriosis research cohort, whereas the GSE87466 dataset was specifically designated for inflammatory bowel disease research cohort. For the validation cohort, GSE23339 GSE126124 datasets were selected. First, patients over 65 were excluded, as well as those with metabolic impairments in both IBD and EM. Secondly, male samples were eliminated from the IBD dataset to remove the influence of gender. Third, blood samples were excluded. Compared to tissue samples, blood samples show more severe distortion in gene expression because blood transcriptomes are not only affected by distorted tissue expression, but also by immune reactions and other tissues in the body. Therefore, in order to prevent errors in blood testing, the analysis of the GSE126124 dataset only includes tissue samples from IBD. **Table 1** shows the dataset specific details. Additionally, a schematic representation of the research design was created (**Figure 1**).

**Table 1:** Sample information for transcriptome data.

GEO Name	Sample size	Control group	Patient group	Platforms	Published
Endometriosis					
GSE7305	20	10	10	GPL570	Apr 09, 2007
GSE23339	19	9	10	GPL6102	May 09, 2011
Inflammatory Bowel Disease(Crohn’s Disease and Ulcerative Colitis)					
GSE87466	108	21	87	GPL13158	Dec 01, 2018
GSE126124	31	9	22	GPL6244	Oct 29, 2019

### 2.2 Identification of differentially expressed genes

During the data preparation phase, limma package (version 4.3.0) is employed within the R environment to conduct a differential analysis on the data. In the process of differential analysis, filtering criteria are set to retain only those differentially expressed genes that satisfied the conditions (| log2FC |) > 1 and P < 0.05.

### 2.3 Functional analysis of differentially expressed genes

The Disease Ontology (DO), Gene Ontology (GO), and KEGG pathway enrichment analysis was conducted by using R packages including ClusterProfiler, org.Hs.e.db, Digure, and EnrichPlot (21). Gene Set Enrichment Analysis (GSEA) was employed to identify significant functional terms between EMS and control samples, plus a GMT reference gene set. Significant enriched terms were determined with a threshold of P < 0.025, a q-value of 0.05, and a false discovery rate < 0.25 (22).

### 2.4 Construction of protein-protein interaction network and hub genes selection

Protein-protein interaction (PPI) networks are utilized to investigate biological processes, including physical interactions, signaling, and metabolic pathways of proteins. These networks aid in comprehending the regulation and function of biological processes in cells, as well as in the identification of crucial proteins. We constructed PPI networks using version 11.5 of the STRING database, which contain shared differentially expressed genes, and screened protein interaction sub-networks with the MCODE tool in Cytoscape (23–25). We used default parameter settings for the screening process (Degree Cutoff: 2, Node Score Cutoff: 0.2, K-Core: 2 and Max Depth: 100). The two topology analysis algorithms(MCC and Degree)in the CytoHubba plugin were used to identify the key genes in the PPI network, which were then visualized using Upset. The Matthews Correlation Coefficient (MCC) is a metric for assessing the quality of predictions in binary classification problems, combining four factors: true positives, false positives, true negatives, and false negatives (26). MCC is able to provide an accurate assessment even in situations of data imbalance. Degree is commonly used in biological network analysis, indicating the connectivity of nodes (27). In protein or gene networks, it reflects how many other proteins or genes are associated with a particular protein or gene. Degree serves as a fundamental representation of network structure and function, where highly connected nodes often represent important biological functions. In PPI analysis, MCC helps to identify core gene groups tightly connected in the network, while Degree reveals the extent of each gene’s connectivity within the network. The common key genes were visualized through Venn diagrams, between the candidate genes from the plugins CytoHubba and MCODE. Finally, the co-critical genes were inputted into the GeneMANIA web tool for analyzing gene co-expression networks (28). The focus was on how these genes interact in EM and IBD, as well as predicting and visualizing genomic functions.

### 2.5 Validation of hub genes for EM and IBD

The diagnostic value of pivotal genes’ expression levels was assessed using ROC (Receiver Operating Characteristic) curves and AUC (Area Under the Curve). The qROC package in R software was utilized to evaluate these genes’ pivotal expression levels in distinguishing between EM and IBD, and to determine their diagnostic ability between the two diseases.

### 2.6 Assessment of the distribution of immune cell subtypes

Once the infiltration of 22 immune cell subpopulations was identified, we used the CIBERSORT algorithm to explore the differences between patients with EM and those with IBD in the control and disease groups. CIBERSORTX is a computational method based on gene expression data that can estimate the relative abundance of different cell types in a sample. CIBERSORTX was chosen because it provides a more accurate and reliable cell composition analysis, which helps to better understand the cellular heterogeneity of the sample (29). Additionally, the abundance and percentage of immune cells in each sample were visually presented.

### 2.7 Immunological correlation analysis

Spearman rank correlation analysis in R was utilized to investigate the correlation between the identified genetic biomarkers and the level of infiltrated immune cells. The identified associations were visualized using graphical techniques from the ggplot2 package,Screening criteria P<0.05 (30).

### 2.8 Identified potential therapeutic small molecule by connectivity map analysis

“Connectivity Map” is a tool or method employed to analyze the correlation between gene expression and drug response (31). It aimed at understanding the connectivity between gene expression and drug effects to uncover potential therapeutic approaches or elucidate drug mechanisms. We input the differential gene expression trends co-expressed in EM and IBD into CMap to identify small molecule compound with potential therapeutic effects. Small molecule compound refer to organic molecules with relatively low molecular weights, typically involved in a biological process as a substrate or product, typically below 900 Daltons. These small compounds could cross cell membranes and are widely used in pharmaceutical and biochemical studies for their potential therapeutic effects. Metabolites identified by CMap are filtered based on enrichment scores (ES, score <-90, P<0.05).

## 3 Results

### 3.1 Identification of differential expressed genes

**Figure 2:**
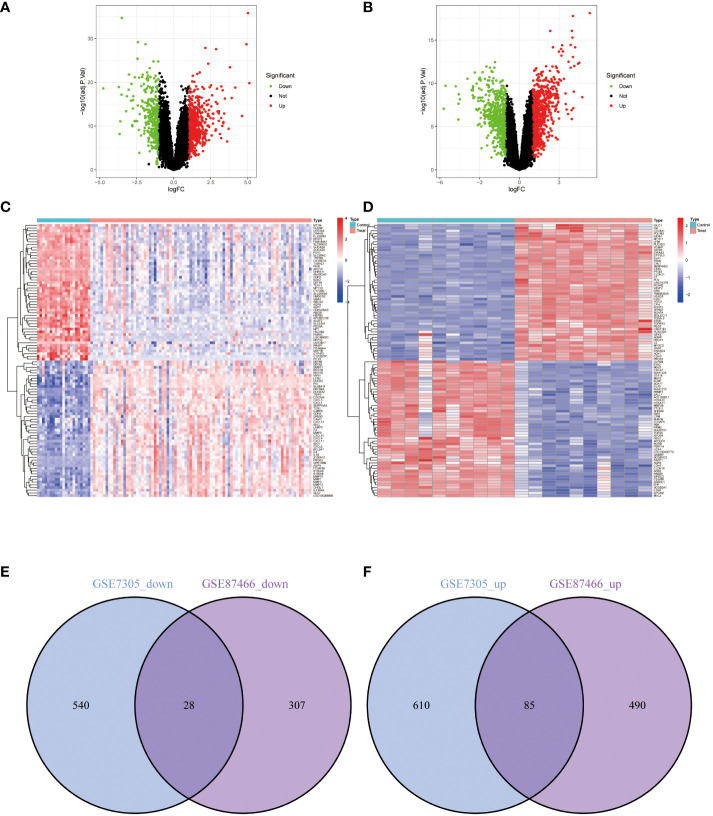
Differentially Expressed Genes (DEGs) Volcano Plots, Heatmaps, and Shared Gene Identification. **(A)** Volcano plot representing DEGs from dataset GSE87466. **(B)** Volcano plot representing DEGs from dataset GSE7305. **(C)** Heatmap representing DEGs from dataset GSE87466. **(D)** Heatmap representing DEGs from dataset GSE7305. Red represents upregulated genes, green represents downregulated genes, and grey represents genes with no differential expression. **(E)** Venn diagram of the downregulated DEGs shared between datasets GSE87466 and GSE7305. **(F)** Venn diagram of the upregulated DEGs shared between datasets GSE87466 and GSE7305.

In the EM GSE7305 dataset, a total of 1263 differentially expressed genes (DEGs) were identified, with 695 up-regulated genes and 568 genes down-regulated genes (**Figures 2A, B**). Similarly, in the IBD GSE87466 dataset, 910 DEGs were discovered, comprising 575 up-regulated and 335 down-regulated genes (**Figures 2C, D**). To assess the overlap of DEGs between the EM and IBD datasets, the Venn diagram R software was utilized, and the analysis revealed that 113 co-expressed DEGs between EM and IBD, including 28 down-regulated genes (**Figure 2E**) and 86 up-regulated genes (**Figure 2F**). These findings imply a potential molecular similarity between EM and IBD.

### 3.2 Functional enrichment analysis of DEGs

**Figure 3:**
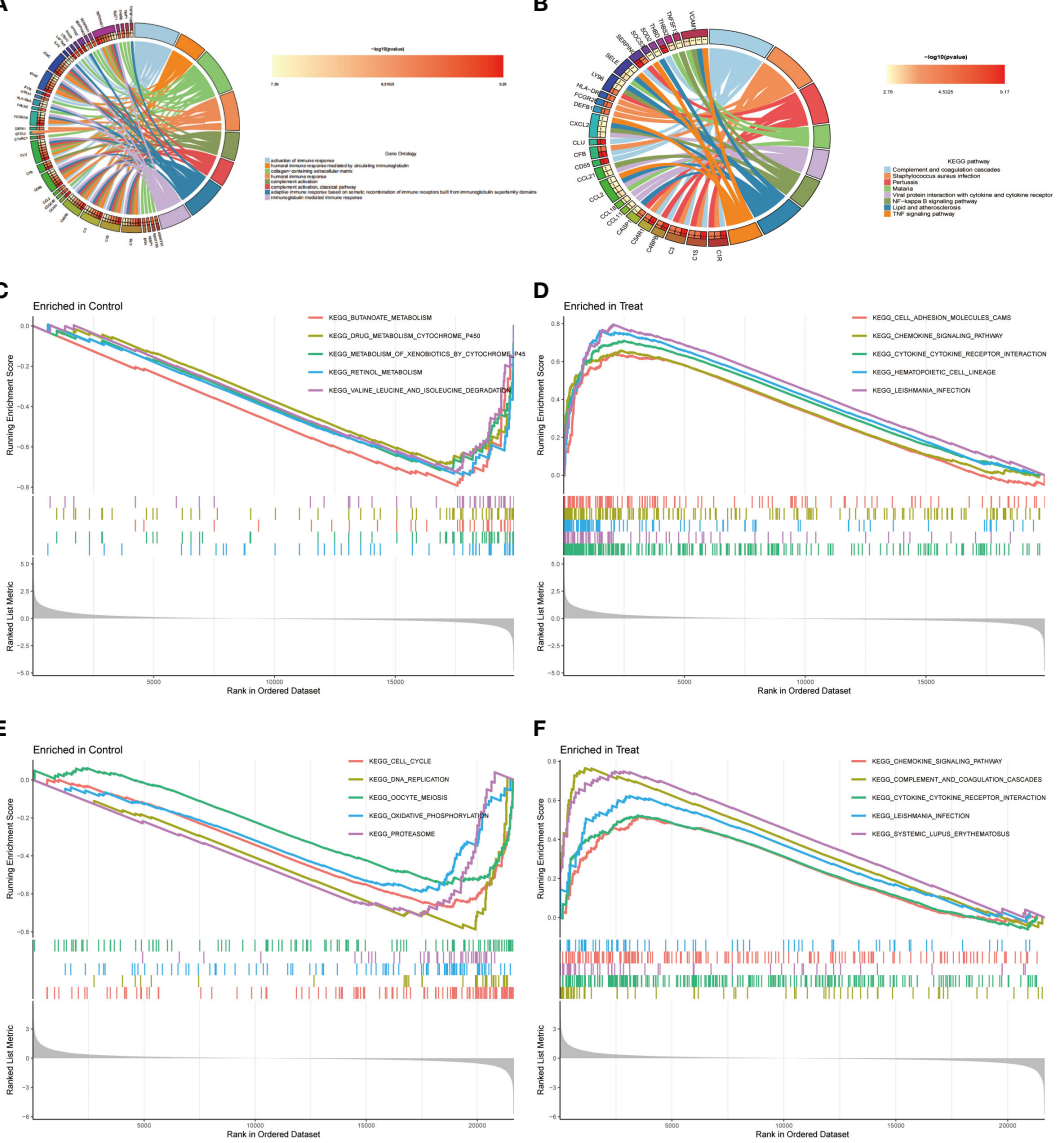
Functional Enrichment Analysis. **(A)** The top 8 items of Gene Ontology (GO) enrichment analysis for shared genes. **(B)** The top 8 items of Kyoto Encyclopedia of Genes and Genomes (KEGG) enrichment analysis. **(C, D)** Gene Set Enrichment Analysis (GSEA) for DEGs between control and disease groups in dataset GSE87466. **(E, F)** GSEA for DEGs between control and disease groups in dataset GSE7305.

The co-expression differential gene of EM and IBD in the functional enrichment analyses focused on three main aspects, which is immune response activation, circulating immunoglobulin-mediated humoral immune response and humoral immune response (**Figure 3A**). Furthermore, the KEGG pathway analysis indicated potential involvement of these differential genes in pathways such as complement and coagulation cascades (**Figure 3B**). The gene expression differs between the diseased and healthy groups. Therefore, we conducted Gene Set Enrichment Analysis (GSEA) to investigate the biological pathways associated with their respective characteristics. The results for IBD are depicted in **Figures 3C–D**, for EM are shown in **Figures 3E, F**.

### 3.3 PPI network and hub genes extraction of common DEGs

**Figure 4:**
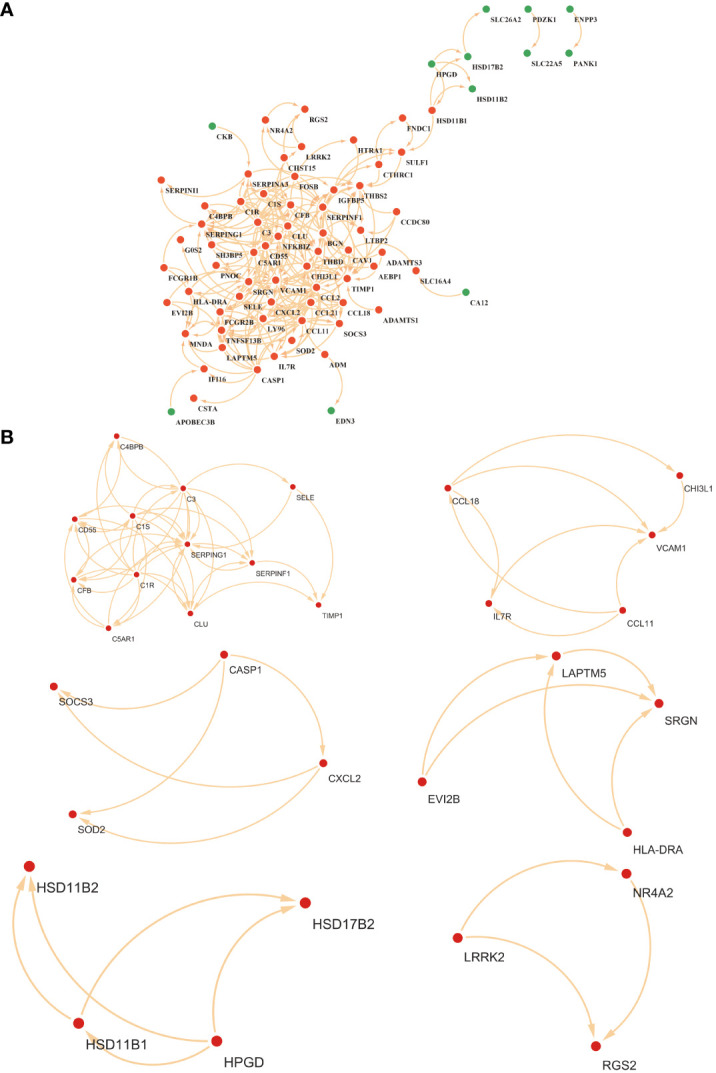
**(A)** Visualization of the protein-protein interaction (PPI) network was constituted by STRING. **(B)** The six key gene modules identified by the MCODE plug-in in Cytoscape.

**Figure 5:**
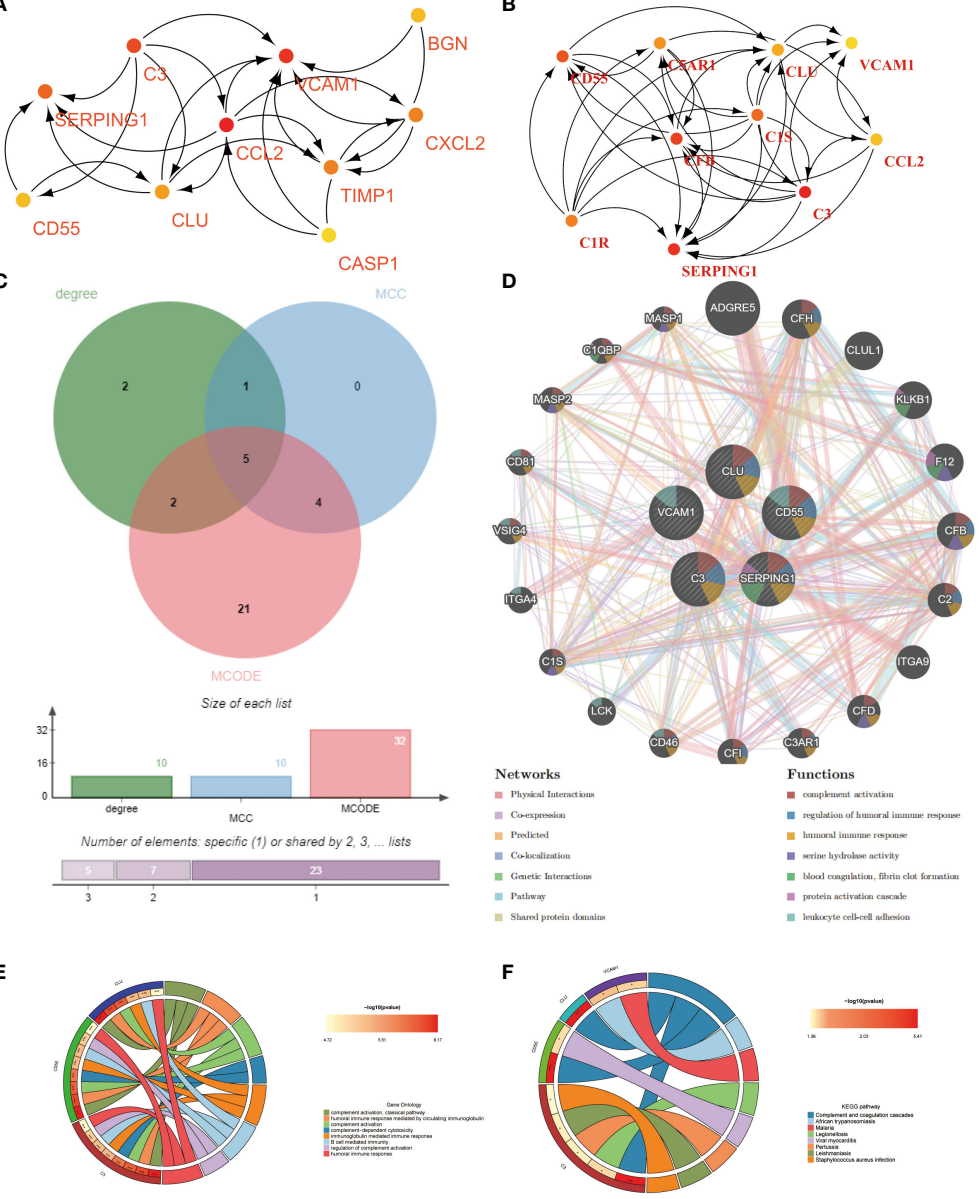
Shared hub genes identification and functional interaction network diagram. **(A, B)** The top 10 core genes identified by Degree and MCC algorithms using the Cytohubba plugin in Cytoscape. **(C)** Venn diagram showing the 5 crossover hub genes between the candidate genes of CytoHubba and MCODE. **(D)** The GeneMANIA diagram illustrates the co-expression interactions between the 5 identified shared hub genes and their neighboring genes. Color codes indicate the functions shared by genes. **(E)** The top 8 items of GO enrichment analysis for shared genes. **(F)** The top 8 items of KEGG enrichment analysis.

The PPI for the differential genes in EM and IBD was carried out using the STRING database (**Figure 4A**). The CytoHubba plugin within Cytoscape software was utilized, employing two topological analysis algorithms, MCC and Degree, to identify the top 10 key genes in the PPI network. Venn plots were used to display 6 core genes identified by both algorithms: SERPING1, VCAM1, CLU, C3, CD55, and CCL-2 (**Figures 5A, B**) respectively. Subsequently, Cytoscape’s MCODE plugin identified 6 core modules, resulting in a total of 32 shared Differentially Expressed Genes (DEGs) (**Figure 4B**). Finally, 5 hub genes were identified using CytoHubba and MCODE visualization plugins (**Figure 5C**). These identified key genes are potentially significant in the pathological processes of both EM and IBD.

Using the GeneMANIA database, we constructed a functional association network demonstrating interactions between genes, where co-expression genes constituted 8.01%, physical interaction genes 77.64%, predicted genes 5.37%, and co-localization genes 3.63% (**Figure 5D**). Gene Ontology (GO) analysis revealed that these hub genes were predominantly enriched in complement activation, classical pathway, humoral immune response mediated by circulating immunoglobulin, and complement activation (**Figure 5E**). Furthermore, KEGG analysis suggested that the hub genes in both EM and IBD might be linked to complement and coagulation cascades, African trypanosomiasis, and the malaria signaling pathway (**Figure 5F**). These findings significantly contribute to a deeper understanding of the potential roles of hub genes in the pathogenesis of these diseases.

### 3.4 Validation of hub genes

**Figure 6:**
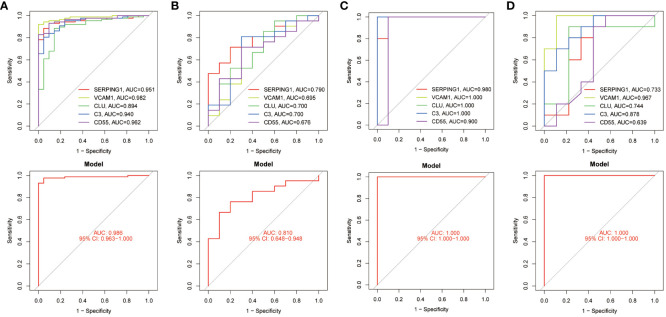
ROC curves testing the diagnostic validity of hub genes in four datasets. **(A)** ROC curve and AUC value for the diagnosis and efficacy validation of hub genes in the GSE87466 test set. **(B)** ROC curve and AUC value for the diagnosis and efficacy validation of hub genes in the GSE126124 training set. **(C)** ROC curve and AUC value for the diagnosis and efficacy validation of hub genes in the GSE7305 test set. **(D)** ROC curve and AUC value for the diagnosis and efficacy validation of hub genes in the GSE23339 training set.

The diagnostic accuracy of the five identified hub genes in both EM and IBD was assessed through ROC analysis. In the IBD GSE87466 dataset, the AUC values for the five hub genes – SERPING1, VCAM1, CLU, C3, and CD55 – all surpassed 0.89, indicating robust predictive power (**Figure 6A**). Similarly, within the GSE126124 training set, the AUC values for these genes were all above 0.67, signifying significant diagnostic potential (**Figure 6B**). In the EM GSE7305 dataset, their AUC values exceeded 0.90, as illustrated in **Figure 6C**. Moreover, in the training set GSE23339, the AUC values for these genes were greater than 0.63, further emphasizing their substantial predictive ability (**Figure 6D**). These consistent and high AUC values from the ROC curve analyses strongly suggest that these 5 common hub genes exhibit robust performance in predicting the risk of both EM and IBD.

### 3.5 Assessment and visualized analysis of the immune infiltration

**Figure 7:**
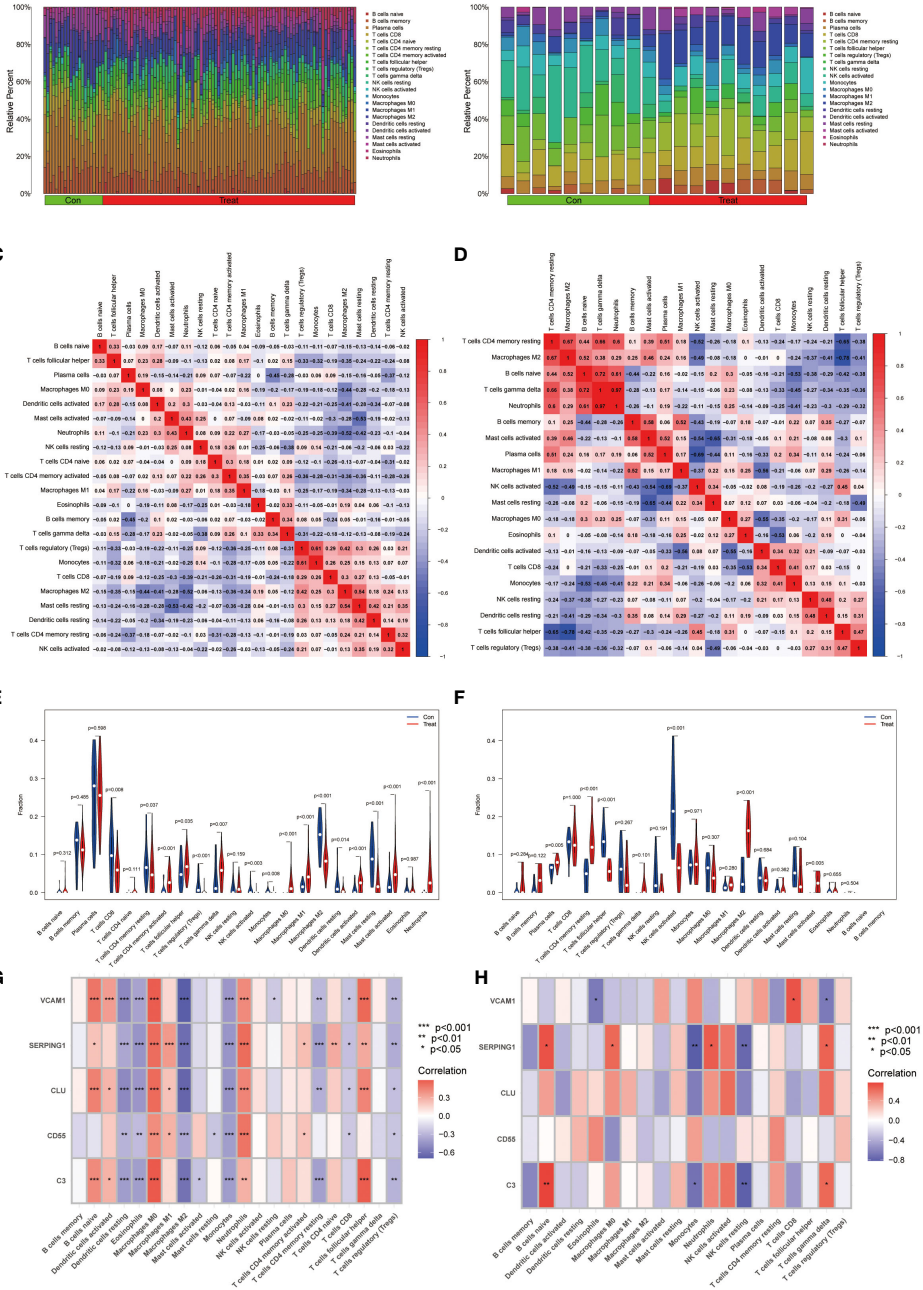
Immunocyte infiltration analysis. **(A, B)** These figures represent the extent of infiltration of various immune cells between the IBD and EM disease groups and the normal group. **(C, D)** Correlation analysis of immune cells in IBD and EM groups. The horizontal and vertical axes represent the names of immune cells, and the values represent the correlation coefficients between immune cells. Red indicates a positive correlation, while blue indicates a negative correlation. **(E, F)** Violin plots depicting the differences in immune-infiltrating cells between the IBD and EM disease groups and the normal group. The horizontal axis represents the names of immune cells, and the vertical axis represents the content of immune cells. Blue represents the normal group, and red represents the disease group. P < 0.05 indicates significant differences. **(G, H)** Correlation analysis of immune-infiltrating cells with genes SERPING1, VCAM1, CLU, C3, and CD55 in IBD and EM.

The analysis of immune infiltration revealed substantial alterations in the immune microenvironment between the diseased groups and the healthy control groups (**Figures 7A, C**). These distinctions were observed in several aspects, including the expression levels of neutrophils, activated mast cells, quiescent mast cells, activated dendritic cells, quiescent dendritic cells, M2 macrophages, M1 macrophages, monocytes, activated NK cells, gamma delta T cells, regulatory T cells (Tregs), follicular helper T cells, activated CD4 memory T cells, and CD8 T cells (**Figure 7E**).

Additionally, significant changes in the immune microenvironment were identified between the endometriosis (EM) disease group and the healthy control group (**Figures 7B, D**). Notably, these groups exhibited marked differences in the expression levels of plasma cells, resting CD4 memory T cells, follicular helper T cells, activated NK cells, M2 macrophages, and activated mast cells (**Figure 7F**). These findings underscore the substantial variations in immune cell composition between the diseased and healthy groups, as well as within specific disease states, shedding light on the intricate immune dynamics associated with these conditions.

In the IBD dataset, we found that the VCAM1, SERPING1, C3 and CLU genes were positively correlated with B cells naive, macrophages M0, activated dendritic cells, neutrophils and T cells follicular helper; VCAM1, SERPING1 and CLU showed negative correlation with resting dendritic cells, eosinophils, M2 macrophages, monocytes, CD4 memory resting T cells and T cells regulatory (Tregs); CD55 was positively correlated with M0 macrophages, M1 macrophages and neutrophils and negatively correlated with resting dendritic cells, eosinophils and monocytes (**Figure 7G**) by correlation analysis.

In the EM dataset, VCAM1 was positively correlated with T cells CD8 and negatively correlated with Eosinophils and T cells gamma delta. SERPING1 was positively correlated with naive B cells, macrophages M0, T cells gamma delta and Neutrophils and negatively correlated with monocytes and NK cells resting. C3 was positively correlated with naive B cells and T cells gamma delta, meanwhile negatively correlated with Monocytes and NK cells resting (**Figure 7H**).

### 3.6 Identification of small molecule compounds of core genes

A total of 9 small molecule compounds targeting therapeutic genes for EM and IBD were identified through the CMap database. The 9 compounds are listed in **Table 2**.

**Table 2:** Results of CMap analysis of the first 15 small molecule compounds.

ID	Name	Description	Score
BRD-K68246049	TTNPB	Retinoid receptor agonist	-97.07
BRD-K32836707	CAY-10577	Casein kinase inhibitor	-96.66
BRD-K49865102	PD-0325901	MEK inhibitor	-95.82
BRD-M07438658	lapatinib	EGFR inhibitor	-94.41
BRD-K64935403	ebelactone-b	Lipase inhibitor	-93.15
BRD-K20285085	fostamatinib	SYK inhibitor	-92.82
BRD-K89014967	AS-703026	MEK inhibitor	-92.58
BRD-K16554956	PTB1	AMPK activator	-90.46
BRD-A01317026	7,8-dihydro-L-biopterin	Dihydroneopterin aldolase inhibitor	-90.38

## 4 Discussion

An increasing number of studies have shown that both EM and IBD involve activation of immune and inflammatory pathways (32, 33). During the progression of EM, specific anti-inflammatory cytokines (IL-1 beta, IL-5, IL-6, IL-7, IL-12, IL-4, and IL-10) and immune cells (M2 macrophage, neutrophil, M1 macrophage, and activated mast cells) are critical in supporting the persistence, expansion, infiltration, differentiation, angiogenesis, and immune evasion of endometriotic lesions in a multitude of pathogenic ways (34–37). The pathogenesis of IBD is closely linked to dysregulation of the immune system. Strober W has confirmed that there is an abnormal mucosal immune response in IBD patients, which may be influenced by both microbiological factors and epithelial cell abnormalities (38). Additionally, inflammation of the intestinal mucosa is intensified by immune cells (T-lymphocytes, B-lymphocytes, macrophages and dendritic cells) and inflammatory mediators (39, 40). There is no doubt that immune cells, inflammatory mediators, and immune-related genes serve as the linchpin connecting between EM and IBD. Our study’s primary aim is to pinpoint shared DEGs in EM and IBD, uncover potential targets. This knowledge will contribute to the improved management and treatment of patients who are afflicted with both EM and IBD simultaneously.

In this study, we screened five hub genes, SERPING1, VCAM1, CLU, C3, and CD55, from a pool of 113 co-expressed genes. GO showed that hub genes were mainly enriched in activation of immune response, humoral immune response mediated by circulating immunoglobulin and humoral immune response etc. The analysis of KEGG showed that these hub genes are primarily involved complement and coagulation cascades, Staphylococcus aureus infection, pertussis and viral protein interaction with cytokine and cytokine receptor pathways. Moreover, GO and KEGG enrichment analyses further highlighted the significant roles of these five hub genes in intercellular adhesion, inflammation and immune pathways. Previous studies have shown that the TLR4-C3 axis can regulate intestinal immune responses during chronic colitis (41). Research byAgostinis C identified the inflammatory feed-foward loop triggered by complement component C3 may serve as a potential therapeutic target for endometriosis (42). Rijcken E et al. studies showed that down regulation of VCAM-1 attenuates leukocyte adhesion and inflammation in IBD rats (43). Thus, these findings collectively suggest the five genes may play important roles in the pathogenesis and development of EM and IBD and may be critical for the regulation of both EM and IBD diseases. The above research findings provide crucial clues for further investigating the connection between these two diseases and potential therapeutic targets.

The SERPING1 gene encodes the C1INH protein, which regulates the immune and coagulation systems by inhibiting the complement system and coagulation cascade to maintain a normal balance of immunity and coagulation (44). Multiomics profiling studies conducted by Yu L et al. revealed a significant increase in the expression of SERPING1 in the EM (L. 45). Additionally, analysis using Weighted Gene Co-expression Network Analysis (WGCNA) has highlighted SERPING1 as a pivotal gene in EM development (46). Furthermore, Lu and colleagues have confirmed the significance of complement activation in the pathogenesis of acute DSS-induced colitis in mice. Their study also confirmed that C1INH treatment suppression of the disease (47). Consequently, aberrant expression or mutations in the SERPING1 gene might be associated with the development and severity of IBD diseases.

C3 gene is pivotal in the immunopathology of IBD, and its faulty activation and regulation may instigate unsuitable inflammatory amplification, resulting in intestinal tissue injury and persistent inflammation. Research has demonstrated that C3 is over-activated in the intestinal tissues of patients with IBD (48, 49). Sünderhauf et al. have proposed C3 as a potential therapeutic target to modulate intestinal immune responses in chronic colitis (41). Moreover, according to Hasan’s Cross-sectional study, C3 serves as an effective serum biomarker for early detection of endometriosis in childbearing-age women with EM (50). research by Agostinis and collaborators has highlighted C3 as a marker for endometriosis, suggesting that its local synthesis might facilitate the implantation of ectopic cysts (42). These findings underscore the significance of C3 in both IBD and endometriosis, emphasizing its potential as a therapeutic target and diagnostic biomarker in these conditions.

CD55 gene is a crucial immune system regulator that primarily functions to constrain the initiation of the complement system. Multiple studies have underscored a significant role for CD55 in diverse disease contexts. An analytical study on bioinformatics suggested that CD55 may act as a potential molecule for a tissue biopsy in ulcerative colitis (UC) (51). Furthermore, immunohistochemical examination have revealed that the expression of CD55 was weak in non-active lesions of UC patients, but significantly enhanced in the inflamed mucosa of patients with active ulcerative colitis (52). In patients with endometriosis, CD55 and other highly expressed cytoprotective proteins guard damaged cells against complement-mediated cytolysis during stress (53). However, the lack of CD55 in the endometrium may result in a rise of endometriotic implants (54). These studies emphasize the significance of the CD55 gene in immune and inflammation-related diseases, providing valuable clues for further research and treatment.

VCAM1 (Vascular Cell Adhesion Molecule 1) is a cell adhesion molecule typically expressed on the surface of endothelial cells. Its primary role is to attract leukocytes to the surface of vascular endothelial cells during episodes of inflammation. Previous research suggests that, while adhesion molecules like ICAM-1 and MAdCAM-1 also contribute to the treatment of inflammatory bowel diseases, selectively blocking VCAM-1 appears to offer higher potential efficacy in managing these conditions. Specifically, this targeted approach significantly reduces disease activity levels and promotes colon health, making VCAM-1 an appealing therapeutic target (55). Additionally, studies have found that blocking ICAM-1 and VCAM-1 can reduce leukocyte adhesion in rat ileitis, indicating their potential in the treatment of intestinal inflammation (43). Moreover, in endometriosis, the expression changes of VCAM-1 and ICAM-1 play a crucial role, and the ratio of soluble VCAM-1 to soluble ICAM-1 may serve as a potential biomarker (56).

CLU,also known as Clusterin, is a secreted glycoprotein. CLU is used as a potential molecular biomarker for diagnosing/prognosticating endometrial proliferative disorders (57). In EM, the expression of CLU is slightly higher in mucous samples, especially in patients who are not using contraceptives. Research has identified CLU predominantly in uterine epithelial and endothelial cells, with the CLU receptor complex primarily distributed in endometrial glands (58). However, currently there have no study been report on the relevance of CLU in inflammatory bowel diseases.

Immune infiltration analyses have unveiled intricate connections between specific immune cells and cell adhesion molecules in both EM and IBD, which could have a notable influence on the development of diseases and associated inflammatory processes. This data enhances our comprehension of the immunological mechanisms in EM and IBD. For instance, the study conducted by Sans and Soriano stresses the significance of blocking VCAM-1 to decrease leukocyte adhesion in rat ileitis and alleviate the inflammatory reaction in IBD (55, 59). Additionally research has highlighted that complement C3 plays a significant role in the inflammatory mucosa of IBD patients, involving the regulation of T cell function. In the mucosa of IBD patients, the expression of C3 and IL-17 mRNA significantly increases and exhibits a strong correlation (41).

In patients with EM, C3 is produced and activated in response to pro-inflammatory stimuli, leading to the activation of mast cells (MCs). These activated MCs exert pathogenic effects on EM by releasing inflammatory factors (42). It has been demonstrated that SERPING1, VCAM1, C3, and CD55 genes in EM and IBD are associated with various immune cells and inflammatory factors. In summary, these findings highlight the importance of the immune system in EM and IBD and suggest that improving therapeutic approaches for these diseases can be achieved by modulating the activity of specific immune cell types and related molecules. These research results provide valuable clues for future treatment strategies and drug development.

Furthermore, based on the analysis of DEGs in EM and IBD, small molecular compounds such as TTNPB, CAY-10577, PD-0325901, and lapatinib were identified through the CMap database, which can potentially reverse the pathological states of EM and IBD. However, this study primarily focuses on exploring the common molecular mechanisms of EM and IBD and providing a potential research direction for the mechanisms of complications associated with these diseases.

This study mainly aimed to investigate potential common pathogenic mechanisms between EM and IBD using bioinformatics analysis. However, the results of the analysis require further biological interpretation and validation. Additionally, these findings may require further functional experiments for confirmation of their biological significance. The study focused on the common pathogenic mechanisms between EM and IBD, but there are other factors, such as genetics and environmental elements, that may influence the development of these two diseases, which may not have been fully considered.

## 5 Conclusion

Five common hub genes (SERPING1、VCAM1、CLU、C3、CD55) have been identified with high diagnostic validity. Enrichment of these genes primarily involves cell adhesion, inflammation, and immune-related pathways. Compared to the healthy control group, both EM and IBD patients exhibit abnormal immune cell infiltration.

## Data availability statement

All expression matrix used in this project can be found in GEO database under the accession numbers GSE7305, GSE23339, GSE87466 and GSE126124.

## Author contributions

HLZ: Data curation, Investigation, Methodology, Software, Validation, Visualization, Writing – original draft. YM: Validation, Visualization, Writing – original draft, Methodology. LW: Validation, Visualization, Writing – review & editing. HZ: Validation, Visualization, Writing – original draft. AS: Validation, Writing – review & editing. SW: Validation, Visualization, Writing – review & editing. XC: Data curation, Investigation, Methodology, Validation, Writing – review & editing. DS: Visualization, Writing – review & editing.
